# Editorial: Early prevention for children with an immigrant background

**DOI:** 10.3389/fpsyg.2022.1106185

**Published:** 2022-12-12

**Authors:** Judith Lebiger-Vogel, Constanze Christina Rickmeyer, Patrick Meurs, Angela Veale, Ilse Derluyn

**Affiliations:** ^1^Sigmund Freud Institut, Frankfurt am Main, Germany; ^2^Faculty of Social Work and Health, Frankfurt University of Applied Sciences, Frankfurt, Germany; ^3^Faculty of Psychology and Educational Sciences, Katholieke Universiteit Leuven, Leuven, Belgium; ^4^Faculty of Educational Science, University of Kassel, Kassel, Germany; ^5^School of Applied Psychology, University College Cork, Cork, Ireland; ^6^Department of Social Work and Social Pedagogy, Faculty of Psychology and Educational Sciences, Ghent University, Ghent, Belgium

**Keywords:** children, prevention, early development, immigrant families, refugee families, caregiver support

This Research Topic sets out to explore developmental challenges children with an immigrant background face within western societies, describing empirically supported and/or relationship-based approaches of early developmental support, in the double sense of the word “early”: as early as possible after the birth of the children or as soon as possible after their migration.

Migration is an essential part of our societies. On top, the current high number of people in forced migration contexts requires extra attention. The integration of these diverse groups is therefore a central social responsibility. Children with an immigrant background are more likely to live in high-risk environments and to experience health and educational challenges. However, it is not their immigrant background as such that puts them at risk, but rather socio-economic and psychological factors associated with their family's migration trajectory. These include complex experiences of loss, unresolved mourning processes, acculturation issues, post-traumatic stress or uncertain future perspectives for refugees as well as everyday racism, low socio-economic status, parental unemployment, insecure residential status, difficult living conditions, etc.

Giving birth to a new generation in migration or exile is a turning point: it can accentuate risk or vulnerability, but can also open windows of opportunities for new directions in people's live. An important question here is how to create environments in which developmental risks can be minimized and adaptive development processes and resilience supported. Experts agree that adaptive development and social integration of immigrant children are optimized by early language or cognitive support and by support of the early parent-child relationship and parenting practices. From longitudinal studies on attachment, we know that children whose early attachment relationships were put under unusual stress—for example, due to difficult experiences in the context of an immigration trajectory—have a greater risk for developing insecure attachment types as well as learning and psycho-social difficulties. Early preventive support can support these children to later develop a better academic achievement, less problems in self-regulation and less other behavioral and emotional problems. In the context of forced migration, post-traumatic stress as a persistent risk factor can also have a pervasive negative impact on children's development processes, whereby they cannot show and develop their best abilities.

Building on these findings, in this Research Topic, one focus is on the earliest possible support in the context of parenting and parent-infant mental health, exploring early preventive interventions, i.e., the First Steps Projects in Belgium and Germany, that aim to create more equal developmental chances for very young children with an immigrant background.

In a conceptual analysis, Meurs et al. describe the challenge of adapting a preventive developmental guidance program in Belgium in a culturally sensitive way: how are different cultural scripts on parenthood and child development integrated in this project, how do different kinds and waves of migration (economic, forced, transnational, etc.) create migration-wave-specific challenges for parents and children?

Lebiger-Vogel et al. report on a randomized control trial for recently migrated mothers and their infants and toddlers in Germany. They show that the effects of a professionally supported psychoanalytically-oriented preventive group method leads to better outcomes, particularly regarding the promotion of emotional availability in mother-child dyads, compared to a similar offer provided by lay persons with a similar migratory experience as the supported mothers.

Meuleman and van Ee explore a group of mothers with children who are born out of sexual violence, a group often hard to reach. What challenges do professionals face in supporting this group? Based on qualitative data, the authors formulate a best practice approach in working with this particular vulnerable group of mothers and children.

Of course, not only young immigrant children face many challenges that may impact their development. Therefore, a second set of papers addresses older refugee and asylum-seeking children and young adults, drawing particular attention to the intersection of culture and developmental pathways. These contributions deal with questions regarding identity processes, experiences of racism and discrimination, self-representations as well as specific challenges of different groups. Especially children with a refugee background, often having experienced war and violence, show a particularly high risk to develop mental health problems.

Spaas et al. describe a school-based collaborative mental health care offer for refugee children in Belgium. By implementing the intervention within a school context, a low-threshold access to mental health care for refugee families is provided. Using findings of an initial exploration of the effectiveness of their intervention, the authors emphasize the importance of interdisciplinary care networks with the involvement of mental health care providers, school staff and the family, whereby sufficient room is provided for family members' culture and migratory experiences.

In a joint Serbian and Norwegian perspective, Varvin et al. explore how developmental tasks, such as restructuring or reorganizing self-experience and identity throughout adolescence, are impacted by forced migration processes and the threatening experiences before and during the flight. These exile-related identity processes lead to specific developmental challenges, whereby this paper expands on the question what health care workers need to know about the unmet developmental needs of these youngsters.

In their systematic review on the effects of everyday racism in adolescent refugees, Metzner et al. show that experienced racial discrimination is a significant predictor for negative outcomes in mental health of adolescent refugees. In contrast, the impact of racism on school-related outcomes is still less clear, due to the lack of empirical studies exploring this association.

At a last contribution in this Research Topic,  Oppedal et al. focus on the specific developmental pathways and positive psychological adjustment processes in unaccompanied refugee youth. Despite increased levels of perceived discrimination and ethnic identity crises among these youngsters, the study provides insights into the stable and high levels of subjective wellbeing in this population. However, subjective wellbeing is negatively associated with perceived discrimination and ethnic identity crises, indicating that interventions promoting subjective wellbeing can decrease acculturation challenges and enable resilient outcomes in these young refugees.

## Author contributions

JL-V drafted the manuscript. All authors reviewed and approved the final version of the manuscript.

